# Authoring Selves in Language Teaching: A Dialogic Approach to Language Teacher Psychology

**DOI:** 10.3389/fpsyg.2022.839152

**Published:** 2022-02-14

**Authors:** Shan Chen, Lawrence Jun Zhang, Judy M. Parr

**Affiliations:** ^1^School of Foreign Languages, Weifang University, Weifang, China; ^2^Faculty of Education and Social Work, University of Auckland, Auckland, New Zealand

**Keywords:** teacher psychology, positive psychology, identity, dialogism, teacher self

## Abstract

The teacher self is a composite psychological construct which encompasses the cognitive, affective, emotional, and social dimensions of teaching. This qualitative study draws on Bakhtin’s concepts of dialogism, answerability, and addressivity to discuss how English language teachers negotiated the shifting and conflictive context to construct selves in relation to the promoted communicative language teaching approach. Based on narrative interviews and classroom observations with five tertiary English teachers in China, we found that these teachers were actively engaged in the *dialog* with their prior learning experiences and active, responsive in answering their contexts while authoring selves in everyday teaching practice. The multiple-case study data support a Bakhtinian understanding that teachers are active users and producers of theory in their own right, highlighting teachers’ agency, creativity, and autonomy. Based on Bakhtin’s dialogism and the case study findings, we bring cognition, identity and practice together and conceptualize the teacher self as having multiple facets and layers: *the autobiographical self*, *the discursive self*, and *the pedagogical self*. The three selves are constitutive of the consummated whole of the teacher self instead of being separate entities functioning individually. The study is concluded with implications for language teacher education and teacher development.

## Introduction

In China, with the accelerated pace of economic development and opening-up, the Chinese Ministry of Education (MoE) launched a new round of curriculum reform nationwide to meet the challenges of globalization. *The Outline of the National Medium and Long-Term Education Reform and Development Plan (2010–2020)* made it clear that the country has an increasing demand for employees with high levels of foreign language competence (Chen et al., 2021; Liu et al., 2021; Wu et al., 2021), in particular, English language proficiency, considering English as a lingua franca (Kecskes, 2019). Thus, a higher bar has been set for foreign language education and a distinct orientation toward developing students’ communicative competence and intercultural literacy can be found in MoE’s goal statements. However, foreign language teaching (mainly referring to English language teaching) in China has been criticized for manufacturing students with deaf and dumb English, or students who are highly competent in English reading and writing but poor in listening and speaking. (e.g., Tsui, 2007; Cheng and Wang, 2012). As *Communicative Language Teaching* (CLT) and *Task-Based Language Teaching* (TBLT) (developed based on CLT principles) are gaining popularity nationwide, more textbooks are published with a CLT orientation. However, debate on the appropriateness of CLT is ongoing. Opponents claim that CLT is not applicable to the Chinese context due to a number of constraints, such as students’ minimal opportunity to use English in real life, large class size, heavy workloads, limited resources, and strong orientations toward exams, all of which pose serious challenges for the teachers to make CLT a reality at the classroom level (Butler, 2011; Liu, 2015). They also point out that communicative approaches are in conflict with traditional Chinese cultures of learning, as they do not provide a well-organized foundation for language learning and they make heavy demands on the teachers (Liu and Jackson, 2009; Liu, 2015; Lu and Ares, 2015). Proponents of CLT, on the other hand, consider CLT as a positive and constructive development in ELT in China and suggest blending memorization and accuracy with communicative activities (e.g., Adamson, 2001; Zhang, 2021). This heteroglossia of language pedagogy places the great majority of Chinese English teachers at the nexus of competing discourses in a postmethod condition (Kumaravadivelu, 2006) with the emergence of a variety of new localized approaches (cf. Wen, 2017; Han, 2018; Zhang and Wang, 2018).

For the frontline language teachers, the choice of an appropriate language teaching approach is crucial and fundamental (see e.g., Liu et al., 2022). The heated political and scholarly discussion and the tapestry of methods and approaches all boil down to the teachers who make the final decisions in the classroom that impact thousands of students’ English learning. Yet teachers’ voices remain underrepresented and empirical studies about how teachers negotiate and make sense of these reform discourses are scarce (for exceptions, see Tsui, 2007; Liu and Xu, 2011). This study was conceived against this backdrop with an aim to examine Chinese College English (CE) teachers’ perspectives through the lens of teacher identity.

## Literature Review

### The Language Teacher Self, Identity, and Cognition

The sense of self or identity comes into play when we interact with another. Everyone performs multiple identities according to one’s understanding as a sense of self in various social roles and contexts (Holzman, 2009; Yuan and Zhang, 2020; Bao et al., 2021). Therefore, the “self” is one of the most heavily researched constructs in psychology, and the study of self has extended beyond psychological domains to attract attention from anthropologists, sociologists and, more recently, over the past 20 years, educational researchers and applied linguists. Despite considerable overlap between *self* and *identity*, Mercer (2016) noted *identity* is highly social, culturally bound and role-based, whereas *self* is characterized by self-concept and self-agency, individuals’ understanding of who they are and what capabilities enable them to achieve. In educational inquiries, the term identity, as with the term self, lacks a clear definition, and is often recognized as the same and used interchangeably with the latter (cf. Danielewicz, 2001; Lauriala and Kukkonen, 2005). While acknowledging the diverse ways of conceptualizing identity and self, we define the teacher identity/self in light of the post-structural view as multiple presentations of self that are exhibited through actions and emotions within various social contexts (Kayi-Aydar, 2015; Zhang and Zhang, 2020).

Identity is found to be intimately related to one’s cognition, such as beliefs, values, attitudes, knowledge (Nagatomo, 2012; Borg, 2015), and emotions (Song, 2016; Wei, 2021). In the fields of general education and language education, interest in teacher identity stems from scholarship in teacher cognition as researchers find that teachers often engage themselves in identity talk when eliciting teachers’ beliefs (Connelly and Clandinin, 2007; Tsui, 2007; Xu and Liu, 2009; Sun and Zhang, 2021). Hence, they argue that teaching is a way of being, “an ongoing process of forming senses of selves in relation to the ways of inhabiting roles and imagined positions that matter to them” (Holland and Lachicotte, 2007, p. 103). Hamachek (1999) poignantly highlights the importance of the identity issue in teaching by saying, “consciously, we teach what we know; unconsciously we teach who we are (p. 209).” In a similar vein, Danielewicz (2001) maintains that good teaching is contingent on identity rather than on ideology or methodology. This line of thinking recognizes teacher identity as a crucial component in determining how teaching is played out. “Identity” thus has become an analytic lens to understand language teachers’ practices and trajectories of professional development (e.g., Tsui, 2007; Liu, 2009; Lee, 2013; Zhang and Zhang, 2015; Jiang and Zhang, 2021; Yuan and Zhang, 2020).

### Identity as an Analytical Lens

Some key issues that define the research agenda in this area include, for example, gender, class, culture, social values, instructional roles, and (non)native-speakerism (e.g., Duff and Uchida, 1997; Farrell, 2011; Nagatomo, 2012; Kayi-Aydar, 2015; Wang and Zhang, 2021). This strand of research, with a focus on the construction of language teacher identity, offers empirical support to the current conception of identity as a fluid, changing, and context-sensitive construct (Zhang and Zhang, 2015; Gao, 2016). Changes in purpose, context, and participant role which are common in social interaction may result in variability of identities. In other words, identity, being a product and a process simultaneously, is socially developed, interpreted, and adjusted according to shifts in the context (Tsui, 2007; Nagatomo, 2012; Zhang and Ben Said, 2014; Song, 2016; Yuan and Zhang, 2020). Understanding selves as teachers thus becomes part of never-ending professional development (Johnson, 2006; Beauchamp and Thomas, 2009).

Inspired by post-structural views of identity, the majority of language teacher identity studies were informed by sociocultural theory, in particular favoring Lave and Wenger’s Situated Learning Theory (cf. Tsui, 2007; Liu and Xu, 2011; Nagatomo, 2012; Yuan and Burns, 2017), which views the process of identity formation as participative, lived experience of belonging and meaning negotiation. To advance the field of inquiry, new theoretical frameworks are desired to enrich further our current knowledge and understanding. This study seeks to fill in this gap by exploring in what ways Bakhtin’s theories of dialogism can be drawn on to understand the dynamic, ongoing process of identity construction of teachers in a discourse-rich reform context.

### A Dialogic Approach to Language Teacher Self/Identity

Bakhtin’s dialogic approach pays attention to voices, or utterances interacting externally with others, the *heteroglossia*, and simultaneously these discourses, when interacting within the self, create a *polyphony* in one’s mind. Therefore, combining a macro (examining the multiple voices present in a specific working context) analysis of the ambiguities teachers face, and a micro (examining the voices within a dialogic self) analysis may reveal not only individual identity struggles and challenges teachers are confronted with, but also the various aspirations teachers attempt to display in being a teacher (Akkerman and Meijer, 2011). For example, Johnston (1997) examined 17 polish English-as-a-Foreign-Language (EFL) teachers’ life histories in an attempt to find out whether they saw themselves as professionals and how they portrayed discursively the meaning of “profession” in their sociocultural context. Johnston’s study took place in 1994 when every aspect of the Polish society was undergoing a post-Soviet transformation. With an explosion of economic activity, in particular trading of exports and imports, EFL increased exponentially. Against this backdrop, Johnston’s attempt to find a shared discourse of professional identity among the polish EFL teachers failed. Rather, he found, drawing on Bakhtin’s discursive approach, teachers’ stories reflected dynamic and non-unitary identities interacting discursively with a range of other discourses from the social, economic, and political context. Professional commitment was seen only in day-to-day terms, as the socioeconomic conditions made it impossible for them to make a long-term commitment. In a similar vein, Menard-Warwick (2011) drew on the concept of heteroglossia to examine Chilean EFL teachers’ engagement with English popular culture (music, video, and the Internet) and how popular culture influenced their decisions on becoming EFL teachers and pedagogy. She found Chilean EFL teachers’ identities and practices were dialogically constructed through individual engagement with English popular culture. Akkerman and Meijer (2011) suggested that the dialogic approach to conceptualizing identity can be helpful in better understanding teacher identity, especially when teachers encounter dilemmas or tensions during their work. However, its heuristic potential has not been fully exercised. We attempt to fill in this gap by addressing the following two questions:

(1) How have Chinese university EFL teachers constructed individual selves in midst of multifarious pedagogical and institutional discourses in local working environments?
(2) In what ways can Bakhtin’s dialogism be drawn to illuminate this ongoing process of dialogic construction of teacher selves?

## Theoretical Framework

### Heteroglossia, Polyphony, and Identity

Bakhtin suggests that the internalization and interplay of social discourses, or *heteroglossia* is the mechanism behind identity development. The heteroglossia, a dynamic interaction of discourses in the world, creates a *polyphony* in the mind, out of which the identity is made. As discourses compete for acceptance and prominence, he suggests that it is through these ideological battles that individual identities are formed. He uses the term *ideological becoming* to describe this identity-making process. Bakhtin’s explanation of ideological becoming is governed by two central concepts: *authoritative discourse* and *internally persuasive discourse* (Bakhtin, 1981). Authoritative discourse is discourse that is enshrined and established. Bakhtin describes it as “religious, political, moral; the word of a father, of adults and of teachers” (1981, p. 342). It is discourse whose “authority was already acknowledged in the past,” “a prior discourse” (1981, p. 342). Authoritative discourse demands our unconditional allegiance. In contrast, internally persuasive discourse is authoritative discourse that has been explored, questioned, and made one’s own. Thus, internally persuasive discourse is a hybrid discourse, consisting of language that is “half-ours and half-someone else’s” (Bakhtin, 1981, p. 345). For authoritative discourse to become internally persuasive, an individual must fill it with his own intention, his own accent, adapting it to his own semantic, and expressive intention (Bakhtin, 1981).

The making of a self, or ideological becoming, unfolds through the transformation of authoritative discourse into internally persuasive discourse. It is through “the process of selectively assimilating” that the individual integrates new social languages into the complex of languages that comprise one’s own identity (Bakhtin, 1981, p. 341). The outcome is a mosaic constructed from pieces of various social languages. Bakhtin’s theorization of how authoritative discourses become internally persuasive discourses provides a distinct perspective to examine the development of teacher beliefs, convictions which are the foundation for identity making. Moreover, Bakhtin’s ideas highlight the central role of discourse, which can be broadly defined as “a pattern of thinking, speaking, behaving, and interacting that is socially, culturally, and historically constructed and sanctioned by a specific group or groups of people” (Miller Marsh, 2002, p. 9), in self-development. His ideas suggest that discourse is the raw material for identity making, and that discursive conflict spurs identity construction and reconstruction. Analyzing the interplay of discourses, thus, can shed light on the work of sense-making and integrating disparate strands of an individual’s identity into a cohesive whole.

### Answerability and Addressivity

Bakhtin (1986) contends that people living in the world are compelled to respond. The state of being and existing is “a state of being addressed and a process of answering” (Holland and Lachicotte, 2007, p. 169). The task of “answering to” others is a significant one for Bakhtin, because it defines an individual’s personhood, state of being, and marks individual identities. Hence, to live means keeping on forming responses to the world (Holquist, 1990). Central to Bakhtin’s two-sided answerability and addressivity is an insistence on engagement in dialogs with others as a way of living and an emphasis on a human’s creative acts and utterances as a form of authoring. In other words, the subject is constantly impelled by the power and authority of its circumstances, while authoring self to respond (Holquist, 2002). Addressivity and answerability are helpful conceptualizations to understand the relationships between teachers and their contexts. As self-authoring subjects, teachers are compelled to form responses and answers to their working environments through which their identities are formed. While recognizing the inherently socially situated nature of human psychology, Bakhtin’s dialogism does not see humans as being solely passive victims of contextual influences. Instead, it views individuals as constructing their own meaning out of contexts and social settings filtered through the lens of their own personal psychology; both of which are “unfinalizable” (Bakhtin, 1984, p. 58), dynamic and open to change across time and place.

## Materials and Methods

### Data Collection

#### Sampling Strategy

This study examines qualitative data that are a subset of a larger mixed methods study of EFL teachers from Chinese universities (Chen, 2017). Teachers indicated their willingness to participate in case studies by filling out a section on a questionnaire. To enable contrast and cross-case analysis, two sampling strategies, typical case sampling and maximal variation sampling, were used to draw five teachers from a pool of 12 potential volunteer participants (Creswell and Poth, 2018). Typical cases, in this study, referred to the different categories of institutions. In maximal variation sampling, the researcher is required to identify certain characteristics or traits to locate individuals that display these different dimensions of the phenomenon, so as to develop multiple perspectives to address the complexity of the research question (Creswell, 2015), so five teachers from three tiers of tertiary institutions were chosen.

#### Interviews, Class Observations, and Artifacts

One-on-one semi-structured interviews were arranged with each participating teacher to probe into their beliefs and identities in narratives (Riessman, 2008), followed by observations of classroom teaching practices. Individual teacher interviews were conducted as three separate interviews spanning several weeks. The aim of initial interview was to create a profile of each participant, their biographical details including their language learning experience and their previous and current teaching experiences. Following the initial interview, the first author observed the practices of individual case study teachers. Individual teachers were observed for two consecutive sessions for a total of 180 or 200 min. Observations were recorded in writing in field notes and each teacher’s practices were recorded on a grid. Post-observation interviews were conducted immediately after the classes when teachers’ memories were still fresh. The grid and field notes were drawn on to stimulate teachers’ recall of instructional activities. In particular, they were prompted to give their rationales for teaching activities as well as for their reactions to students’ performances or responses. When dissonance between articulated beliefs/identities and practice were noticed (e.g., in Ellen’s and Jessie’s cases), additional questions were asked to further probe into the causes. All interviews with teachers were conducted in Chinese to eliminate any barrier created by using a foreign language. Both interviews and classroom observations were audio recorded and transcribed for analysis.

To enrich and triangulate data sources, the first author also viewed and copied webpages of the three institutions’ CE Departments which provided relevant information about the program of CE teaching, the promoted approach to teaching and assessment, updates of on-going reforms, research and teacher development programs. On a voluntary basis, participating teachers shared photocopied pages of the textbook, lesson plans of the observed unit, and students’ writing samples (Tony and Sunny). These artifacts were employed as supplementary data to enhance understanding of observational notes and the participants’ narratives in the interviews.

#### Participants

All five teachers are Chinese, non-native speakers of English, working in three different tiers of institutions. Pseudonyms were used to maintain the anonymity of research participants. Tony and Sunny worked at a tier-1 institution, Nancy and Jessie in a tier-2 institution, and Ellen in a tier-3 institution. All teachers were capable of using English as the medium of instruction. A brief biography for each participating teacher is provided below. Tier 1 institutions are national key universities receiving funding primarily from MoE. Universities belong to Tier 2 are provincial tertiary institutions which are mainly supported by the local governments. Tier 3 institutions are smaller in scale and offer a limited range of programs. Tier 1 and 2 institutions usually attract top students with high matriculation scores while Tier 3 institutions are at disadvantage in recruiting high academic achievers. Students enter tertiary level education with varying levels of English competence and motivation, and their prior learning experiences and strategies also differ since they come from different parts of China with uneven economic and educational development. Demographic information of five participating teachers is summarized in Table 1.

**TABLE 1:** Case study teachers’ profiles.

Teacher’s name (pseudonym)	Gender	Background (u-undergraduate, m-master’s, d-doctorate)	Teaching experience	Overseas experience	Classes being observed
Tony	Male	English (u) linguistics (m)	6 years	yes (2 years)	Reading
Nancy	Female	English (u) American Literature (m) Applied linguistics (d)	7 years	yes (2 years)	Listening and speaking
Sunny	Female	Marketing (u, m both in English-speaking countries)	5 years	yes (4 years)	Integrated English
Jessie	Female	English (u) American Literature (m) Applied linguistics (d)	12 years	yes (2 years)	Reading and writing
Ellen	Female	English education (u & m)	19 years	no	Integrated English

Tony: Tony began his teaching career as a CE teacher in a national key university, T University, after graduating from a prestigious normal university with a master’s degree in Applied Linguistics and Language Teaching. Prior to taking up this full-time position, he had tutored at private language schools for IELTS training, so teaching English has been his specialty since then. Tony had been through two rounds of curriculum reform. CE was once taught as an integrated course and then was broken down to two big chunks (reading and writing, and listening, and speaking) and, when the study took place, the department offered extension courses specifically for advanced learners, such as *British and American Cultures* and *Practical Reading*. Tony was teaching the *Practical Reading* class when this study took place, and he had previous experience of teaching reading, writing, listening, and speaking at T University.

Nancy: Nancy’s institution, S University, is a Tier 2 university. Nancy graduated with a master’s degree in English and American Literature from this university. Due to her academic excellence, she was offered a teaching position by her university to become a CE teacher. She has spent most of her time teaching English Listening and Speaking to freshmen and sophomores in this university. At the time of the present study, it was her 7th year of teaching the Listening and Speaking classes. According to the placement test scores, her students were at the basic level.

Jessie: Jessie worked at the same institution as Nancy with the same group of students but she was responsible for the teaching of Reading and Writing. She has been teaching CE for 12 years. Her bachelor’s and master’s specialty was American Literature. Jessie was working on her Ph.D. part-time at the time of study.

Sunny: Sunny had taught CE in T University for 5 years. Sunny has a bachelor’s and a master’s degree in Marketing and has studied for 4 years in New Zealand and United Kingdom. Sunny’s academic background was distinctive from other participants because traditionally EFL teachers in China are required to have a bachelor’s degree in English or English Education.

Ellen: Ellen’s university, Y University, is a Tier 3 institution. Ellen had worked in her institution for 19 years, so she had been through the school’s transformation from a city college to a provincial comprehensive university. At the time of study, Ellen was an associate professor in the Department of College English. Ellen graduated from one of the top national normal universities with a bachelor’s degree in English and a master’s degree in English Education. After graduating, she took up a teaching position in Y College, which later became Y University.

### Data Analysis

Qualitative data analysis is a continuous, iterative process in which data condensation, display, and conclusion drawing/verification were interwoven before, during and after data collection (Miles and Huberman, 2013). Analysis of qualitative data began upon researchers’ entering the field and continued throughout the process of data collection. Similar to Grossman’s (1990) three-level analysis, the first level of qualitative data analysis focused on individual cases, aiming to provide an in-depth portrait of each teacher. The data generated from the first round of interviews and observations were transcribed and analyzed to identify teachers’ beliefs, identities, and practices to create such profiles. For example, Tony identified himself as an inspirational teacher, a facilitator, whose priority was to motivate students to use English for communicating ideas and to broaden their horizons through learning English. The second level involved processing data holistically examining the three dimensions embedded in stories: temporality, sociality, and place (Barkhuizen, 2008). At this stage, we scrutinized teachers’ stories to examine teachers’ ideological becoming over time, particularly how they made sense of the promoted CLT approach as an EFL teacher within their peculiar intermediate working contexts, and individual ways of responding to competing discourses in teaching. Taking Jessie’s case as an example, the temporal dimension of her stories revealed that her endorsement of CLT grew out of her own negative learning experience with TA. However, within her work environment (place) and broader institutional and social contexts (sociality), she experienced difficulties in translating her orientation toward CTL into practice due to a range of contextual constraints.

The third level of data analysis went further and involved cross-case analysis across contexts. Exploration of similarities and differences and identification of patterns proceeded during the process. For instance, all teachers held negative views on traditional grammar-translation approach by describing it as test-oriented (Tony, Jessie, Ellen, and Sunny) and not functional (Nancy). After they entered teaching, they endorsed CLT because it is functional (Nancy) and ideal (Ellen). At this stage, Bakhtin’s concepts were drawn on for an in-depth understanding and interpretation of the data. Conceptualizing the CE teacher’s mind as a polyphony containing multiple discourses and voices competing for supremacy, we observed that teacher self is multifaceted, and three layers of self, that is the autobiographical self, the discursive self, and the pedagogical self, figured prominently in teachers’ stories, interwoven and integrated to constitute a fluid, dynamic, and complex teacher self. The three levels of data analysis and their foci are summarized in Table 2.

**TABLE 2:** Three levels of data analysis and focus of analysis for each level.

Stage	Focus of analysis
Thematic analysis	Identifying themes related to beliefs and identities, features of teaching practice, tensions and conflicts in cognition and practice, ways of responding to conflicts.
Structural analysis	Examining temporality, sociality, and place in stories.
Cross–case analysis	Identifying patterns and interpreting data with the theoretical framework.

During the data collection and analysis processes, the first author transcribed interviews and observations, assigned emergent codes to data, and developed themes with reference to the key concepts of the theoretical framework; while the second and third author were involved in checking, refining codes and interpretations to ensure the consistency of both the process and the product. This “inquiry audit” suggested by Lincoln and Guba (1985, p. 317), was an important measure to enhance the dependability of qualitative research. Furthermore, participating teachers were invited to review the transcripts and comment on preliminary results for member checking, an important technique for establishing credibility in qualitative research (Lincoln and Guba, 1985: Creswell and Poth, 2018).

## Findings and Discussions

In this part, we draw on Bakhtin’s concepts of dialogism, answerability, and addressivity to discuss how teachers have assimilated CLT-oriented beliefs, how they have negotiated the context to develop self-identities, and how they have authored selves in teaching practice. Through the three levels of analysis, we found the three facets of teacher self, namely, the autobiographical self, the discursive self, and pedagogical self, are inextricable and deeply rooted in teachers’ appraisal of past learning experiences, constantly engaged in dialogs with one’s personal histories and contextual discourses and subject to change and revision as new discourses emerge.

### The Autobiographical Self: Dialogizing With Personal Histories

Teachers were actively engaged in the *dialog* with their prior learning experiences in the form of what Bakhtin called “internal speech” (Bakhtin, 1984, p. 238). Episodes of their personal learning histories, their encounters with the language, language teachers, and various language pedagogies entered teachers’ inner speeches or consciousness. These experiences and encounters provide the ground for developing values, opinions, beliefs, and a sense of self.

Throughout their language learning journeys, these teachers had the opportunity to expose themselves to traditional approaches (TA) as well as CLT. TA was the former authoritative discourse that dominated ELT in China for four decades whereas CLT emerged as a new norm and mandate, a new authoritative discourse guiding ELT in modern China. As they experienced the different methods as learners or teachers, they gradually developed an evaluative stance toward these approaches. For instance, Nancy, in Excerpt 1, compared CLT and TA, and found CLT was more effective in developing learners’ competence in the use of the target language.

Excerpt 1

“In China, teachers follow a textbook, whereas, in France, they don’t even have a textbook to guide instruction. However, students have ample opportunity to speak/practice around a particular language point. They encourage efforts in making up your own sentences to express ideas. You are not required to imitate templates or sample sentences. It is okay to make mistakes and you do not need to feel bad about it; while in the Chinese classrooms, you are required to memorize sentence patterns you probably will never get the chance to use. Unlike in the Chinese classrooms, utterances produced in the French class are highly relevant to realities. In contrast, Chinese students who can recite a big chunk of text, when confronted with a real-life situation, do not know how to respond. They can recite from memory but can’t talk. The Chinese students are trained to be parrots who can repeat and recite but if you change the wording, they don’t know how to respond” (N-INT-1-29∼32).

“It is a shame on the Chinese English language teaching, as a whole, and a huge frustration on the students, to see that after more than 10 years of learning, students are still not able to use the language for daily conversations” (N-INT-2-1∼5).

Nancy was able to acquire French fluency in her 2-year visit in French, and she attributed her success of learning a third language to function-based CLT adopted in her French classes. She then came to embrace CLT and intended to create opportunities to engage students in communicative tasks in her own English classes after she returned to work. Along with Nancy, Tony, and Sunny also expressed explicitly that they did not enjoy being taught with the grammar-focused, teacher-centered approach at secondary schools, and they concluded it was only instrumental for preparing them for tests and exams. Such unpleasant experiences prompted them to seek a viable alternative. CLT, which advocates meaningful communication-based, student-centered methods, met these teachers’ expectations and thus was accepted by them. Through reflecting upon and evaluating episodes in their stories of English learning and teaching, theorizing them with newly acquired professional knowledge and practices, they were able to create new meanings out of old stories. In other words, they selectively appropriated both TA and CLT discourses and their understanding of the theories is populated with their own intentions and their own voices (Bakhtin, 1981).

From a Bakhtinian perspective, this dialogic interaction between the self and its personal history is open-ended, ongoing, and “unfinalizable” (Bakhtin, 1984, p. 58) charged with creative potential. Being responsive and answerable to the autobiographical self is generative to creativity and vitality in teaching. Including Nancy, three teachers were found to be actively engaged in dialogizing with their prior learning experiences through which new meanings were negotiated and generated. For example, Sunny recalled throughout her years of English learning experience as a student in China, none of the teachers ever explained to her *how* English can be effectively learned, and this has thrown her into confusion and compelled her to seek effective ways of learning English. This learning experience has now become the major principle to guide her teaching practice: focusing on *how* (the context, collocations, text organization, and ways of thinking in English, and the connection between them) language can be efficiently acquired rather than *what* (knowledge about words and grammar) should be memorized.

Previous research has shown that teachers’ experience as learners has a key influence on teachers’ cognitions thereby shaping instructional practices (e.g., Grossman, 1990; Xiang and Borg, 2014). However, shaping is a one-directional act in which the former gives a definite form of the latter. In Bakhtin’s view, one-sidedness, or being monologic, leads to stagnation and dying out. Instead, the dialogic interaction or double-voicedness implies a two-sided meaning exchange. Lortie (1975) identified teaching based on imitation as apprenticeship of observation, as opposed to individual decision making. Such apprenticeships of observation can be helpful to get novice teachers started, but it can lead to potential stagnation in professional development. Based on the case study data, we contend that teachers are able to engage in ongoing, unfinalizable dialog with their own learning histories and lived experiences, which contributes to sustained professional development and learning.

### The Discursive Self: Dialogizing With Multiple Social and Contextual Discourses

**FIGURE 1:**
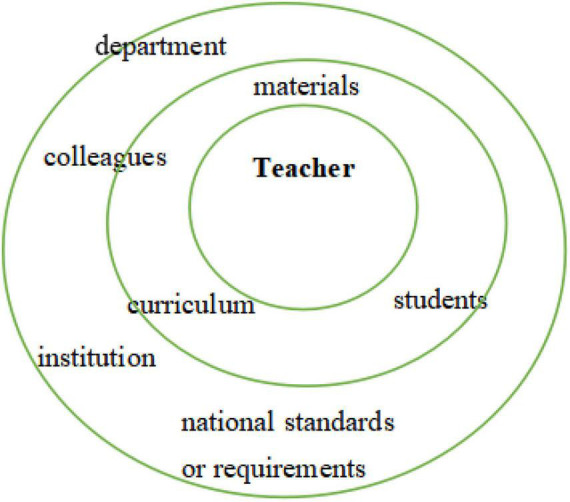
Matrix of plural discourses enclosing the teacher.

Teachers were active and responsive in answering their contexts. The *discursive self* is constructed through interaction with multiple social discourses and forces (Miller Marsh, 2002; Beauchamp and Thomas, 2009). Discursive selves characterize the way teachers speak and talk as well as how they act and react in particular social situations. The discursive self is the product of a particular sociocultural era. A teacher’s articulated teaching philosophy, pedagogical choice, and relationship with students and others are embodiments of the discursive self. The interview data demonstrated that the five participating teachers, coming from three levels of tertiary institutions, were all surrounded by a complexity of multiple contextual discourses. Apart from the inner voices coming from within each teacher, they were also compelled to listen to and respond to the voices of their students, their departments, institutions, materials, or textbooks, the curriculum and its requirements, and the broader social context outside of universities. The heteroglossia of multiple social discourses with each representing a different ideological system often causes a cacophony in self rather than a coherent harmony (see Figure 1 and Table 3). In the following excerpts, Sunny explained how students’ traditional learning habits kept her from practicing CLT.

**TABLE 3:** Heteroglossia of contextual discourses.

Contextual discourses	Traditionalism	CLT
College English curriculum requirements (2007)		+
Social political rhetoric		+
Institutional context	+	
Departments (policy making)		+
Departments (lack of support and training)	+	
Students (motivated, high proficiency)		+
Students (unmotivated, low proficiency)	+	
Textbook (CLT-oriented)		+
Textbook (traditional)	+	

*+ indicates the particular factor in favor of the corresponding teaching approach.*

Excerpt 2

“I used to focus on the text, no emphasis on vocabulary or grammar. Later on, I found students tend to refer to the glossary to study vocabulary. If I don’t go through the new words with them, the words are like strangers to them as if they have never met them in class” (S-INT-1-14∼18).

Excerpt 3

“We played a vocabulary game to check their vocabulary. The students knew the words but were not able to sound them out. Later, I found out the reason: this group of kids did not have a listening section in the university entrance exams, so their English classes focused solely on silent reading and writing [deaf-and-dumb English] at the secondary school. They were not able to comprehend what I was saying in the class. When I said “spice,” they had no idea what I was talking about. I realized the gravity of the problem and decided to bring the tape to the class and have them hear the pronunciations and read after the tape. It’s not what I wanted to teach in College English but I can’t ignore it” (S-INT-6-2∼12).

Sunny’s internally persuasive discourses about language teaching included a focus on language function and context which was in alignment with CLT. While feeling confident about her own position, Sunny was surrounded by a number of disabling discourses which drew her away from teaching communicatively. The story of Sunny making compromises to accommodate students’ learning habits with respect to vocabulary was a case in point. This non-communicative learning had significantly changed the nature of her CLT-oriented pedagogy by absorbing much of the time she originally planned for communicative tasks. Figure 1 illustrates a matrix of social and contextual factors enclosing and confronting Chinese tertiary English teachers, and each represents a discourse that is oriented toward either TA or CLT (see Table 3).

Another frustrating discourse that featured in teachers’ accounts across three levels of institutions is students’ lack of motivation for English learning and CE being an increasingly marginalized course in universities. For example, T University was going to reduce the credit hours for CE by 50%. Students were expected to take CE only in their freshman year. This change, from Tony’s perspective, was suggesting to the students that English was not as important as the major courses. Likewise, in Jessie’s class, she was constantly frustrated by students’ indifference to her English class. Jessie commented:

“If you ask them to preview the content, only a few will do so. Sometimes, I would have to make a rule and let them know that I will check their preparation before class begins, but how can I find out if everyone has done it and the thoroughness of their preparation? You can only pick a couple of students and get a rough idea of whether they have done their study by asking questions. I found that some students are too much occupied with their major studies. By the way they answered my questions, I can tell who has previewed and who has not. Some students are highly motivated, and they actively participate in the class, whereas others always make me frustrated because no matter how diligently I urge them to do their work, they won’t listen. I talk to them and explain the importance of studying English. They understood it without any problem, but they just won’t do it (J-INT- 2-26-30).

In Ellen’s institution, in which students’ English abilities are generally well below those of students in Tony’s and Jessie’s classes, Ellen encountered the same issue. She mentioned, “When the content is difficult and they can’t understand it in English, students lose their interest; even if I taught the basic grammar in Chinese, some students were still not interested. I was really puzzled” (E-INT-1-46).

Teachers’ awareness of such influences and their ways of responding to them constitute the formation of a teacher identity, and it has been found from the five teachers’ stories that teachers’ metacognition and positioning strategies varied significantly when facing such a heteroglossia. Tony and Nancy revealed themselves as advocates for the communicative teaching approach, and they consistently positioned themselves as such in the way they talked about and enacted their identities. Although they were tempted to lean toward the traditional approach by the contextual constraints, such as the required allegiance to textbook and the large class size, they strove to work against all odds to create conditions and opportunities for CLT to uphold a communicative orientation of their lessons. On the contrary, Jessie and Ellen’s ways of responding were submissive; in spite of intentions to teach communicatively, their sense of helplessness and resignation was profound, so they allowed the traditional discourse to take the upper hand. The following excerpt illustrates Ellen’s resignation.

“To be honest, I think my teaching approach remained very traditional like high school English teachers, not like a university English teacher. The communicative approach did not work. It was difficult to carry through with CLT because students were not able to respond. I tried group work. I had four students work as a group, but they were not able to perform the discussion task. The foreign teachers demonstrated group work with us (English teachers), and it worked because the teachers were more linguistically capable. I think the students’ English abilities were the major obstacle” (E-INT-2-9∼14).

### Authoring Self and Pedagogy: The Pedagogical Self

The last facet of the teacher-self accentuates the language teacher’s autonomy, agency, and creativity. The pedagogical self is the way by which teachers define their space of teaching and pedagogy in their own terms as answerability and establish authorship within their social contexts. The development of the pedagogical self is a dialogic and creative process of analyzing a context and interpreting it with our own words, or *active responsive understanding* (Bakhtin, 1981), and the self is viewed as self-defining entity fused with the possibility of authorship (Holquist, 2002; Vitanova, 2010). In this sense, one can define agency as one’s ability to transcend what is given and create something new, particularly through the combination of unrelated discourses, the invention of words and concepts, or the imagining of alternatives and possibilities (Davies, 2000). An essential element of authorship is active engagement with one’s situation as well as the distinctiveness of one’s responses: answerability/responsibility and emotional-volitional tone (a complex of one’s feelings, desires, and moral evaluations) (Bakhtin, 1993; Vitanova, 2005).

Tony’s, Nancy’s, Sunny’s, and Ellen’s narratives and teaching practices exemplified the everyday authorship and creativity of Bakhtin’s answerability. To be specific, Tony’s design of the Book Flood activity enabled him to transform the solitary reading act into a communicative literature circle that facilitated students’ reading comprehension and communication of meanings. Nancy integrated cultural knowledge and learning into the teaching of vocabulary and everyday English to connect English with students’ real life and stimulate their interest. Sunny experimented with the process writing approach and used translation for contrastive analysis to help students better understand the linguistic differences between the two languages. Ellen’s story sharing and improvised teaching is also a variation of pedagogical creativity that enabled her to bond with students and helped her to discover the rewarding part of teaching.

These teachers’ creative acts and adding a personal touch to their established teaching methods empowered teachers to resolve deftly epistemological and contextual tensions present in autobiographical and discursive selves and, ultimately, contribute to positive psychology, and emotional well-being (Wang et al., 2021). Exemplifying this are the following excerpts.

“I think the students like my class because I teach the language in the context and connect it with cultures. It’s my style” (Nancy: N-INT-10-20∼23).

“I have been to the United Kingdom, so when the lesson has a part involving the United Kingdom, I will tell them what I have experienced, and students loved that. It’s a motivator for students because they feel it is fun and interesting. Once they become interested in the foreign cultures, they realize they have to learn English to know more about them, so it creates an internal and instrumental drive for English learning that goes beyond the classroom” (Nancy: N-INT-2-8∼13).

“I think my teaching is more of an inspiration and stimulation. I teach English by inspiring students. That is who I am, my style of teaching” (Tony: T-INT-1-19∼20).

“I told my students that they made me fall in love with my job again, and my passion and love is increasing every day. I enjoy communicating and interacting with students. The learning outcomes of my students no longer affect my motivation, though I get upset if sometimes they do not make desired progress” (Ellen: E-INT-4-35∼40).

The pedagogical self focuses on how teachers reveal themselves as teachers, their ownership of the content, ideas, and voice; and their means of establishing authority in everyday teaching activities. This pedagogical self or *self as an author* can be considered as an extension of the autobiographical self in that the teacher’s inner dialog with his or her life history may have generated ideas to create something new. It is also intimately tied to the discursive self in that consciousness, voices, and behaviors are all situated and context-specific in order to have a meaning, so it is impossible to separate acts from thought or the social context.

**FIGURE 2:**
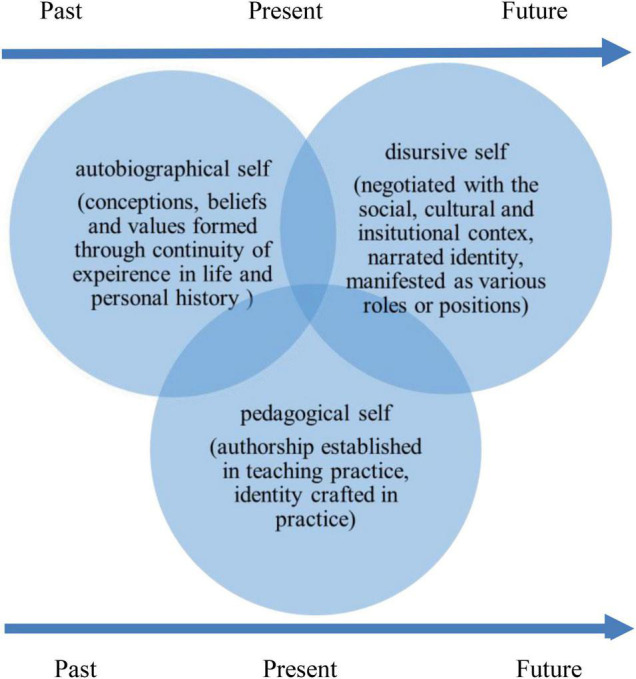
The three selves: a holistic interactional model.

Taken together, we propose a holistic interactional dialogic framework to examine and understand language teacher identity and psychology (Figure 2). The autobiographical self acknowledges that teachers came to teaching with their own personal histories which provided the base for developing beliefs and conceptions of ELT. When the autobiographical selves are met with teaching contexts which contain an array of variables, such as students, the curriculum and the institutional context, teachers are faced with a heteroglossia, simultaneously entering into a dialogic space requiring them to negotiate with each of the variables. The discursive selves are therefore constructed through negotiating and renegotiating with these variable discourses. The discursive self emphasizes that teachers’ teaching practices are highly situated activities, a result of dialogic interactions and negotiations of meanings. Surrounded by a complexity of multiple discourses, teachers are compelled to position themselves in relation to the social discourses, as well as respond to them. The pedagogical self reflects how individual teachers respond to the context through authoring their personhood and everyday acts in EFL teaching. The three selves constitute the whole language teacher identity that is taking form gradually through chronotopic construction, a synchronized performance of multiple historicities (Blommaert and De Fina, 2016), and is future-oriented, unfinalizable, dynamic and open to change across time, and space.

## Conclusion and Implications

Consisting of five case studies, this research examined individual teachers’ conceptions and the ways in which they managed to live with competing discourses through constructing an identity that fused the multiple layers of self and through creating a self-authoring space. We summarize the key findings that emerged from the qualitative data as follows: (1) The stories and experiences of the participants indicated teachers construct selves and identity through selectively appropriating authoritative discourses, transforming them into internally persuasive ones and negotiating with a range of competing discourses. (2) The rich data support a Bakhtinian understanding that teachers are active users and producers of theory, in their own right (Johnson, 2006). Identities are not something teachers *have* or *are* but a resource that they *use* or *claim.* (3) A dialogic approach to language teacher self views individuals as constructing their own meaning out of contexts and social settings filtered through the lens of their own personal psychology; both of which are unfinalizable, dynamic and open to change across time and place.

Though our study focused on EFL teachers in Chinese tertiary institutions, our discussion of the multiple selves and the holistic interactional model is relevant to any reform settings in which participants are experiencing dramatic changes with new ideas, technologies or modes of working and communicating introduced or mandated. A holistic view of teachers’ cognition, practice and identities, drawing on Bakhtin’s notions of dialogism, answerability, heteroglossia, authoritative discourse, and internally persuasive discourse, highlights teachers’ subjectivities and agency. Bakhtin’s philosophy of the self as an author, whose existence pivots on creative answerability, allows disempowered individuals to transcend their subjective position. Hence, language teachers are no longer conceived as being locked in fixed social positions as passive recipients of social order as suggested in many previous teacher cognition studies (cf. Farrell and Kun, 2008; Phipps and Borg, 2009; Xiang and Borg, 2014). Rather, teachers as creative Bakhtinian subjects are capable of authoring their own consciousness, utterances, and acts through dialogic activities.

This study also offers significant implications for teacher education programs and teacher professional development. First, examining language teachers’ experiences as language learners and teachers can contribute to a more nuanced understanding of their views, agencies, and identities. Identifying and understanding conflicts, competing voices, and *sites of tension* in their stories is critical to addressing the curricular challenges teachers face today (Kayi-Aydar, 2015; Aoyama, 2021; Jiang and Zhang, 2021). Second, to improve EFL teaching, it is necessary for institutions, departments, and teacher development programs to help teachers invest in “identity capital” (Matthys, 2012, p. 59) to become self-authoring knowers, or, in other words, to increase self-awareness of their own beliefs, roles, and practices as well as the underlying reasons behind their pedagogical decisions. Institutions and departments as shareholders can serve as catalysts to facilitate this process. It is recommended that faculty meetings or teacher development programs provide opportunities for dialogical exchange and personal reflection to help teachers develop self-regulating capacities, including the capacity to reflect on their multiple roles; to construct theories about their relationships, and to understand how the past, present and future relate to each other. As a result, they will empower teachers to demonstrate their competency, achieve their goals, and work toward their highest potential.

## Data Availability Statement

The original contributions presented in the study are included in the article/supplementary material, further inquiries can be directed to the corresponding author.

## Ethics Statement

The studies involving human participants were reviewed and approved by the University of Auckland Ethics Committee on Human Participants. The patients/participants provided their written informed consent to participate in this study.

## Author Contributions

SC conceived the study, collected and analyzed the data, and drafted the manuscript. LZ finalized it for submissions as the corresponding author. All authors revised the manuscript and contributed to the article and approved the submitted version.

## Conflict of Interest

The authors declare that the research was conducted in the absence of any commercial or financial relationships that could be construed as a potential conflict of interest.

## Publisher’s Note

All claims expressed in this article are solely those of the authors and do not necessarily represent those of their affiliated organizations, or those of the publisher, the editors and the reviewers. Any product that may be evaluated in this article, or claim that may be made by its manufacturer, is not guaranteed or endorsed by the publisher.
